# Supplementary material for: Co-Occurrence of Staphylococcus aureus and Ochratoxin A in Pasteurized Milk
Source: Toxins (Basel). 2022 Oct 21;14(10):718. doi: 10.3390/toxins14100718 (PMC9612031; doi:10.3390/toxins14100718)
Supplement: Supplementary file 1 [file toxins-14-00718-s001.zip › toxins-1916153-supplementary.pdf]

Table S1. Detection of Staphylococcus aureus in pasteurized milk. Data from December 2014 to September 2015. Unit is CFU/mL.

	Jan	Feb	Mar	Apr	May	Jun	Jul	Aug	Sep	Oct	Nov	Dec
Fengtai District	6	9	7	7	8	50	15	33	24	2	3	8
Daxing District	8	4	7	6	6	40	4	22	13	2	7	8
Shijingshan District	8	7	6	7	7	40	7	24	15	6	4	9
West District	7	4	5	5	5	8	14	11	13	4	1	10
East District	3	5	9	6	6	55	27	41	34	2	3	3
Chaoyang District	4	4	6	5	5	30	5	18	11	2	4	9
Tongzhou District	4	6	5	5	5	18	8	13	11	5	4	3
Changping District	2	5	6	4	5	100	16	58	37	7	10	8
Shunyi District	2	9	9	7	8	60	20	40	30	3	5	15
Haidian District	3	7	6	5	6	39	80	60	70	6	6	13

Table S2. Detection rate of bacteria in pasteurized milk.

Sample Name	Total number	*Staphylococcus aureus*	*Coliform*	*Enterobacter sakazakii*	Total number of bacterial colonies
UHT milk（A、B、C brands）	360	-	-	-	10.56%
Pasteurized milk(C brand)	120	99.17%	15.83%	2.50%	97.50%
Pasteurized milk(D brand)	90	91.11%	28.89%	13.33%	97.78%

“-”: not detected.

Table S3. OTA detection level of each district in each month in pasteurized milk. Data from December 2014 to September 2015. “-”: not detected.

	Jan	Feb	Mar	Apr	May	Jun	Jul	Aug	Sep	Oct	Nov	Dec
Fengtai District	0.46	-	-	-	-	0.7	0.17	-	-	12.4	-	-
Daxing District	0.46	0.32	-	-	-	-	-	-	0.11	-	-	-
Shijingshan District	-	-	-	-	-	1.11	-	-	-	11.1	-	-
West District	-	0.46	-	-	-	-	0.51	0.44	-	-	0.188	0.95
East District	-	-	-	-	-	-	-	-	-	9.94	-	-
Chaoyang District	0.26	0.36	-	-	-	0.26	0.51	0.29	-	-	0.436	18.8
Tongzhou District	-	-	-	-	-	0.38	-	-	-	-	-	-
Changping District	-	0.21	-	-	-	-	-	-	-	-	-	16.5
Shunyi District	-	-	-	-	-	0.77	-	-	-	4.74	-	15.2
Haidian District	0.17	-	-	-	-	-	-	-	-	5.36	-	7.02

Table S4. Detection of OTA in UHT milk and Pasteurized milk.

Content(μg/kg)	UHT milk		Pasteurized milk	
	**number**	**proportion**	**number**	**proportion**
<1.0	51	14.17	20	16.67
1.0-5.0	24	6.67	2	1.67
5.0-10.0	4	1.11	3	2.50
>10	1	0.27	5	4.16
Total	80	22.22	30	25

AFM1 was not detected in 120 pasteurized milk. AFM1 was detected in two samples of 360 UHT milk, and the detected amounts were 0.27 μg/kg and 0.16 μg/kg, respectively.
